# Transmissibility of emerging viral zoonoses

**DOI:** 10.1371/journal.pone.0206926

**Published:** 2018-11-07

**Authors:** Joseph W. Walker, Barbara A. Han, Isabel M. Ott, John M. Drake

**Affiliations:** 1 Odum School of Ecology, University of Georgia, Athens, Georgia, United States of America; 2 Center for the Ecology of Infectious Diseases, University of Georgia, Athens, Georgia, United States of America; 3 Cary Institute for Ecosystem Studies, Millbrook, New York, United States of America; 4 Southeastern Cooperative Wildlife Disease Study, College of Veterinary Medicine, University of Georgia, Athens, Georgia, United States of America; National Taiwan University, TAIWAN

## Abstract

Effective public health research and preparedness requires an accurate understanding of which virus species possess or are at risk of developing human transmissibility. Unfortunately, our ability to identify these viruses is limited by gaps in disease surveillance and an incomplete understanding of the process of viral adaptation. By fitting boosted regression trees to data on 224 human viruses and their associated traits, we developed a model that predicts the human transmission ability of zoonotic viruses with over 84% accuracy. This model identifies several viruses that may have an undocumented capacity for transmission between humans. Viral traits that predicted human transmissibility included infection of nonhuman primates, the absence of a lipid envelope, and detection in the human nervous system and respiratory tract. This predictive model can be used to prioritize high-risk viruses for future research and surveillance, and could inform an integrated early warning system for emerging infectious diseases.

## Introduction

Zoonotic viruses pose major threats to human health [1–5]. These viruses, which emerge from animal reservoirs, can cause epidemics that require substantial resources for containment [6, 7] if sustained human-to-human transmission occurs. Even limited outbreaks from stuttering chains of secondary (human to human) transmission can overwhelm local health systems and undermine social and political stability [4, 8, 9]. Recent outbreak responses have been largely reactive, rather than preemptive, often resulting in inefficient action and costly delays [9]. Developing a more proactive strategy for spillover prevention will require novel predictive tools [10].

Animal viruses must pass through a series of highly selective evolutionary bottlenecks to become established in the human population [11]. First, the host species barrier selects for viruses that establish successful infections in exposed humans [11, 12]. The next bottleneck selects for viruses capable of relatively efficient human-to-human transmission. This efficiency can be measured by the pathogen’s basic reproduction number, *R*_0_, defined as the mean number of secondary infections propagated by an initial case in an immunologically naïve population. Selection for variants with higher *R*_0_ values produces viruses capable of more sustained transmission within the new human population [12]. During this process, two discrete thresholds can be defined: first, whether or not an animal virus can infect humans, and second, whether or not a zoonotic infection can be transmitted between humans. To supplement knowledge on the first threshold, animal-to-human spillover, several data-driven models have been used to predict undiscovered zoonotic viruses, potential vectors [13, 14], and animal reservoirs [15–17]. With regards to the second threshold, a descriptive statistical analysis by Geoghegan *et al* found that human transmissibility was generally associated with low host mortality, chronic infection, non-segmented genomes, and the lack of an insect vector [18].

In this study, we use a predictive machine learning model to identify specific virus species that may have undocumented potential for human-to-human transmission. The tree-based machine learning method we employ in this analysis, known as gradient boosted regression trees, offers a number of distinct advantages over more parametric statistical models. Boosted regression trees accommodate diverse data types and are robust to hidden interactions, missing data, and co-linearities among variables (e.g., correlated viral traits that arose through shared evolutionary history) [19, 20]. Additionally, a machine learning approach also allows us to model and interpret complex nonlinear relationships between predictor variables and the response, which can be obscured in parametric regression models. Finally, these methods identify viruses that best fit the trait profile of a zoonotic virus with secondary transmission in humans, giving more precise targets (particular viruses, or viral clades) for surveillance and future research.

## Results

**Fig 1:**
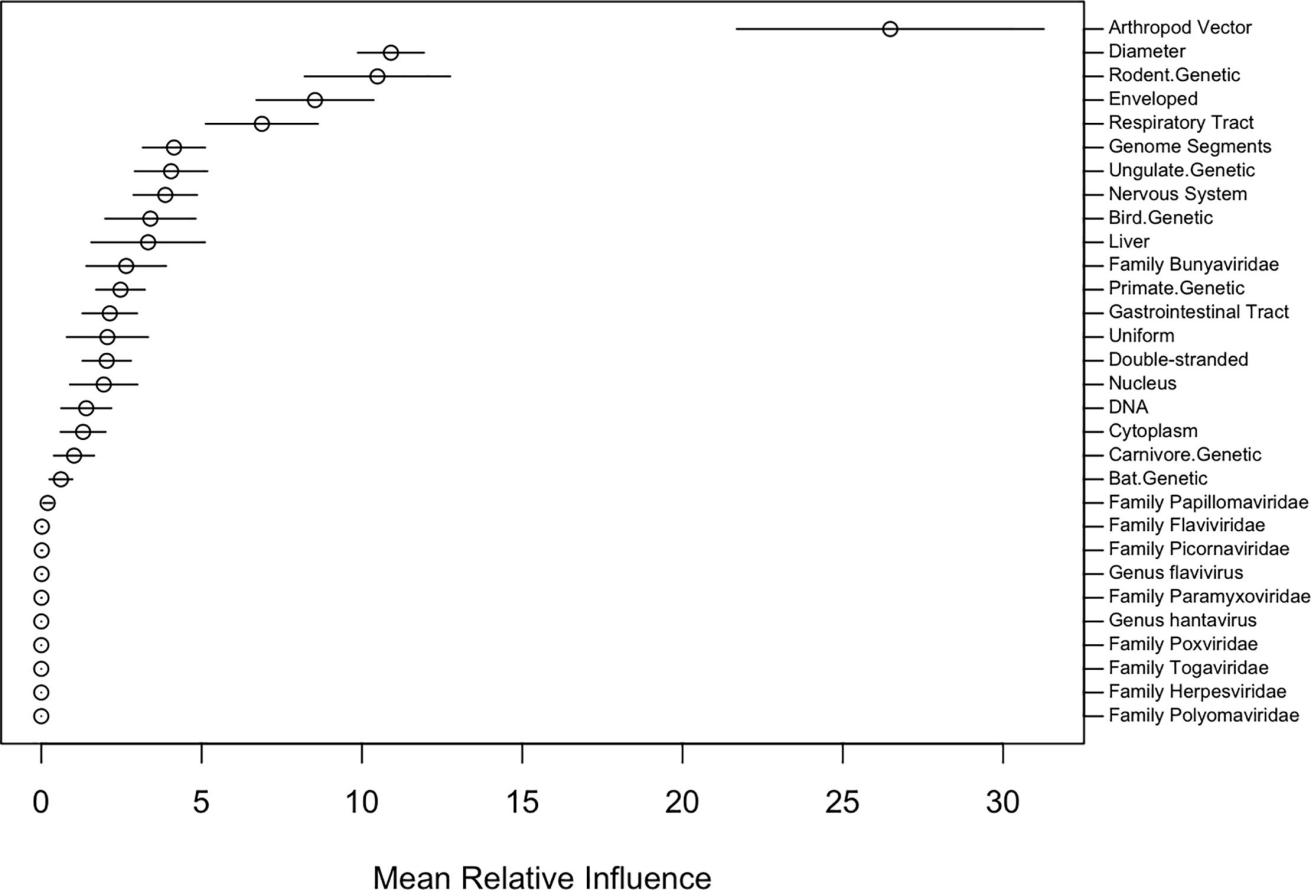
Relative influence of predictors, averaged across 40 models. For each of the 40 boosted regression tree models in our primary ensemble, the normalized relative influence of each predictor variable was computed using Friedman’s algorithm ^45^. This figure shows the average of these scores (mean relative influence) for each predictor variable in our dataset that was included in at least one model of the ensemble (mean relative influence > 0). Horizontal lines represent the interval formed by ± 1 standard deviations. Exact relative influence values are listed in S2 Table.

**Fig 2:**
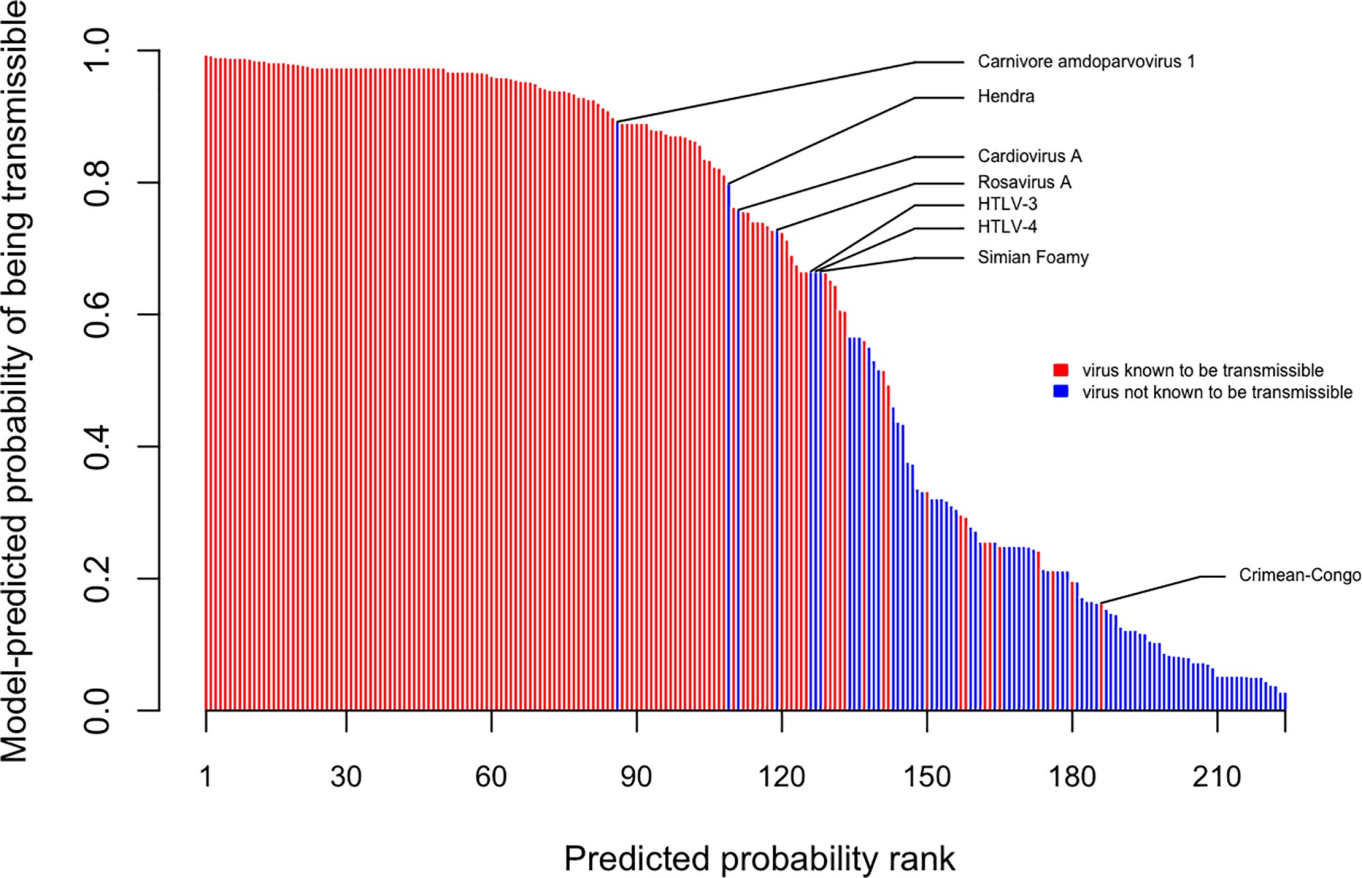
Predicted viral risk index. This figure contrasts the observed transmission ability of all 224 viruses in our dataset (red = human-to-human transmission observed, blue = human-to-human transmission not observed) with their average model-predicted response probabilities, as assigned by the primary boosted regression tree models. This model ensemble accurately discriminates transmissible and non-transmissible viruses, as illustrated by the lack of “overlap” of the two groups in the rank-ordering. The highest ranked viruses that are not currently known to be transmissible between humans were Carnivore amdoparvovirus 1, Hendra virus, Cardiovirus A, Rosavirus A, Human T-lymphotropic viruses 3 & 4 (HTLV-3/4), and Simian Foamy virus. Crimean-Congo haemorrhagic fever virus was the lowest ranked species for human-to-human transmission has been documented.

**Fig 3:**
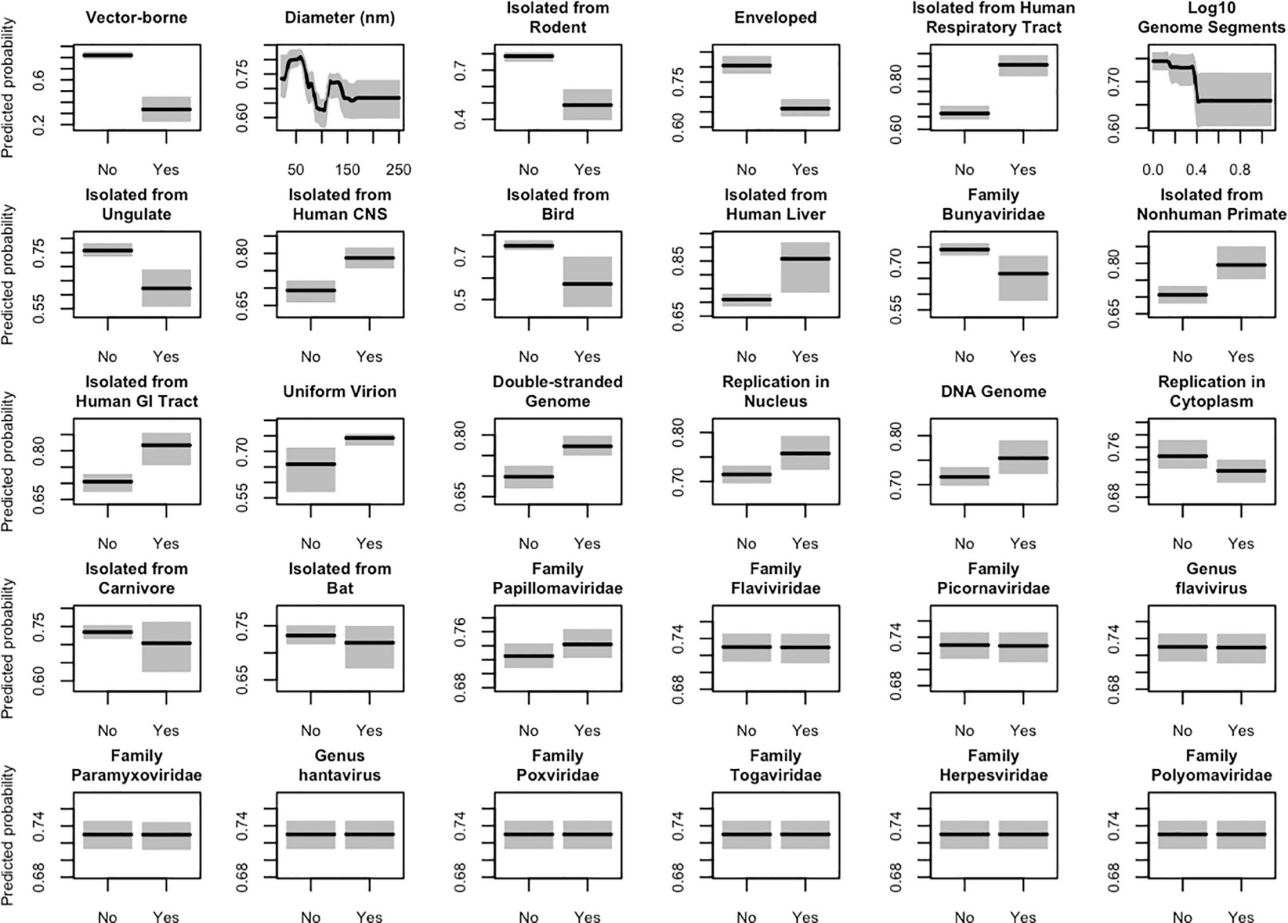
Variable partial dependence plots. Partial dependence plots show how the model-predicted probability that a virus is able to spread between humans is affected by individual viral traits when the effects all other predictors are controlled for. Dark lines represent the median predicted transmission probability across the 40 boosted regression tree models of the primary ensemble, while shaded regions represent the corresponding 95% confidence interval. Viral features are ordered by their mean relative influence within the primary boosted regression tree models from left to right, then top to bottom. Predictor variables with a mean relative influence score of 0 are not included in this figure. Trait definitions and exact relative influence scores are given in S1 and S2 Tables, respectively.

Consolidating records from the primary literature and existing pathogen databases yielded a list of 224 virus species known to infect humans. For each virus, we collected data on taxonomic grouping and 19 biological features (S1 Table), 16 of which do not have a counterpart in previous analyses [18, 18]. We then assigned each virus a binary score denoting whether or not it was known to be transmissible between humans; non-transmissible viruses received a score of 0, while viruses transmitted directly, via an arthropod vector, or through environmental contamination received a score of 1. Fitting boosted regression trees to this data produced an ensemble of models that identify strong predictors of human transmissibility (Fig 1) and accurately discriminate transmissible from non-transmissible viruses: among zoonotic viruses, models distinguished those with observed secondary transmission with ~84% accuracy (median AUC = 0.8430 +/- 0.0778); when applied across all human viruses (zoonotic and non-zoonotic), the model achieved even higher accuracy (median AUC = 0.9196 +/- 0.0353), demonstrating that zoonotic viruses with secondary transmission are easily distinguishable from all other human viruses on the basis of observable viral traits (Fig 2). AUC scores, tuning parameters, and the relative influence of covariates are reported in S2 Table. We used partial dependence plots to characterize the relationships between predictor variables and transmissibility in our model (Fig 3).

Our models identified certain non-human hosts of zoonotic viruses as strong predictors of secondary human transmissibility. Zoonoses carried by nonhuman primates were more likely to be transmissible between humans than other zoonotic viruses, while viruses found in rodents and birds were less likely to be transmissible. Arthropod-borne viruses also showed a significantly reduced probability of being transmissible between humans compared to directly transmitted viruses, corroborating previous findings [18]. Only 13.21% of the arboviruses in our dataset were also transmissible between humans, compared to 76.61% of non-vector-borne viruses.

Virus presence in certain human tissues was also predictive of human transmissibility in our model. Virus detection in the liver, the central nervous system, and the upper and lower respiratory tracts was associated with an increased likelihood of transmission between humans. The respiratory tracts support successful viral entry and establishment, as they place a large surface area of exposed mucosal membrane in direct contact with the environment [21, 22]. As the livers and central nervous systems of living patients are relatively inaccessible to diagnosticians, false negatives among under-sampled viral zoonoses could bias the observed relationships between viral presence in these locations and transmission ability.

A number of structural features were also associated with human transmissibility, including the absence of a lipid envelope, small virus particle size (< 75 nanometers (nm) in diameter), and limited genome segmentation (≤ 2 segments). These relationships have been identified in a previous analysis [18]. Non-enveloped virions are often more stable in the extra-host environment than enveloped particles, and can therefore remain infectious in the air, in water, and on surfaces for longer periods of time [23]. This corroborates previous findings [18]. The elevated stability of non-enveloped viruses could partially explain the association between smaller virion size and human transmission in Fig 3, since the lipid envelope contributes to the diameter of the virion; in our dataset, the average diameter of enveloped and non-enveloped viruses was 109 and 52 nm, respectively. The negative relationship that we observed in our models between number of genome segments and transmission likelihood does not contradict Geoghegan *et al*, which classified genome segmentation as a binary variable and found that segmented viruses are collectively less likely to be transmissible between humans [18].

Although many viral features, including genome length and strandedness, are phylogenetically conserved, only 11 of 104 (10.6%) binary family and genus variables were estimated to have non-zero relative influence in any model, with a combined mean relative influence of only 2.66% (S2 Table). We did not observe significant changes in AUC scores or the trait profile after removing taxonomy variables from the model (S2 Table), suggesting that the covariates in our model capture the majority of the important family- and genus-wide traits that underpin secondary human transmission.

Using our primary model ensemble, we ranked viruses by their mean predicted probability of being transmissible between humans (Fig 2 and S3 Table). Of the 85 human viruses not currently known to spread between humans, 47 were assigned higher probabilities than Crimean-Congo hemorrhagic fever virus, the lowest ranked species with known transmissibility between humans. Within this subset, the viruses with the highest probability of secondary human transmission were Carnivore amdoparvovirus 1 [24], Hendra virus [25], Cardiovirus A [26, 27], Rosavirus A [28], HTLV-3 and 4 [29], and Simian Foamy virus [30]. These pathogens may be predisposed to developing human-to-human transmissibility, and some may already be capable of transmission between humans, as underreporting and misdiagnosis of infections often allow viruses to spread unobserved [31]. Future epidemiologic studies of these pathogens should make efforts to identify potential human-to-human transmission.

To determine the potential influence of study bias, we created a separate ensemble of models, trained on the same data-splits as the models in our primary ensemble, that included the log-transformed number of PubMed citations for each virus as a predictor, a measure of research effort. Study effort was relatively influential in these models (mean relative influence = 20.685, 2nd highest of all predictors), but its inclusion did not improve overall predictive performance, and the rankings of the most influential variables were highly similar between the two ensembles (Kendall rank-order correlation = 0.929). Comparing partial dependence plots between the two ensembles shows that including study effort as a predictor does not meaningfully alter the predicted trait profile of transmissible viruses (S1 Fig). Furthermore, rankings of individual virus species by their predicted transmission probability were similar between models that do and do not include study effort, with a Kendall rank-order correlation of 0.86. These comparisons suggest that the primary model ensemble is not severely confounded by study bias.

In this analysis, viruses capable of spreading between humans directly, environmentally, or through an intermediate arthropod vector are labeled as “transmissible.” To assess the robustness of our findings to different definitions of the “transmissible” label, we fit a separate ensemble of GBM models with a modified response variable definition. For these models, we labeled viruses as “transmissible” (binary response = 1) if they are capable of spreading between humans directly or through the environmental, while viruses that are not known to spread between humans at all or require an intermediate vector to spread are classified as “non-transmissible” (binary response = 0). This change in definition had the effect of reclassifying the response of 5 arbovirus species (chikungunya, yellow fever, dengue, o’nyong-nyong, and Oropouche) from “transmissible” to “non-transmissible”. The rankings of predictors by mean relative influence were almost identical between ensembles (Kendall rank-order correlation = 0.985), and a comparison of partial dependence plots shows that the trait profiles of directly transmissible viruses and transmissible viruses as a whole do not significantly differ (Fig 3 and S2 Fig).

Not all viruses were accurately classified by our model. In particular, some viruses with known human-to-human transmissibility were assigned a low predicted transmission probability (Fig 2). The 10 human-transmissible viruses with the lowest predicted response values belong to 5 families: *Bunyaviridae*, *Togaviridae*, *Arenaviridae*, *Flaviviridae*, and *Poxviridae* (S3 Table). These families contain relatively high numbers of individual virus species, few of which are known to be transmissible between humans (S3 Fig). Further molecular characterization of these viruses may identify factors that distinguish human-to-human transmissible “outlier” viruses from their non-transmissible relatives.

## Discussion

These results are subject to two key qualifications. First, by considering viruses at the species level, our approach does not account for variation in human transmissibility within individual species. For example, the H1N1 subtype of Influenza A has caused several human pandemics [32], whereas the H3N8 subtype within the same species primarily infects dogs and horses, and has never been isolated from humans [33]. Second, our approach does not consider the efficiency of observed human transmission per se, and handles viruses with supercritical (R_0_ > 1) and subcritical (0 < R_0_ < 1) transmission identically. Future research aiming to preempt novel viral spillover events will depend on building mechanistic understanding at these smaller scales.

In this study, we show that the human-to-human transmissibility of zoonotic viruses can be predicted with a high degree of accuracy by ensembles of machine learning models trained on observed viral features. This data-driven modeling framework could allow public health workers to broadly characterize the epidemic risk posed by novel viral pathogens. Linking these models with those trained to predict the zoonotic capacity of animal viruses [15] could provide a data-driven method for focusing broad-scale virome sampling projects.

Our model ensemble predicts that zoonoses carried by nonhuman primates are more likely to be transmissible between humans relative to other zoonotic viruses, while viruses found in rodents and birds are less likely to be transmissible. This corroborates current theories on viral emergence, which posit that both phylogenetic and physical proximity between human and animal hosts drives spillover events and the success of subsequent adaptations [34–36]. Closer phylogenetic relationships between hosts generally correspond to physiological and molecular similarities that allow secondary transmission in a new host species to occur with less extensive viral adaptation [2, 3, 12, 34, 36]. In contrast, for viruses that infect phylogenetically distant hosts, beneficial adaptations to one host system can significantly reduce pathogen fitness in the others, impeding the evolution of transmissibility within the new host population due to adaptive trade-offs [34, 36, 37]. However, close physical proximity can increase contact rates between humans and more distantly related host species, creating additional opportunities for spillover. While genetic dissimilarities may initially inhibit the development of secondary transmissibility in humans, increased contact creates more opportunities for successful viral adaptation [21, 38].

The negative association between arthropod vector infection and human-to-human transmissibility in our model ensemble suggests that arboviruses, none of which exclusively infect humans, are significantly constrained by the evolutionary trade-offs needed to infect both phylogenetically divergent arthropod vectors and vertebrate host species [34, 36, 39]. Viruses that overcome this barrier most commonly circulate between nonhuman primate hosts and anthropophilic vectors, again illustrating the importance of phylogenetic and physical proximity in successful viral emergence [2, 34, 36]. In contrast, the vast majority of arboviruses infect genetically dissimilar organisms via zoophilic vectors, typically only infecting humans living in close proximity with reservoir hosts. Humans are predominantly dead-end hosts for such viruses.

Few quantitative studies have investigated the associations between viral traits and human transmissibility. Our findings corroborate the results of one such study (Geoghegan *et al* [18]) in several important ways: both analyses found that DNA-based genomes were associated with human transmission, while genome segmentation, presence of lipid envelopes, and associations with arthropod vectors predicted dead-end spillover. Beyond these physical attributes, our model ensemble also shows how the human-to-human transmissibility of viral zoonoses is shaped by ecological and evolutionary context, with virus isolation from non-human primates and the human respiratory tract and CNS being positively associated with human transmission (Fig 3). In our model ensemble, the variables describing the animal hosts and human organ systems from which viruses have been isolated together account for over 38% of the overall variable influence (Fig 1, S2 Table).

We demonstrate that a highly predictive model ensemble based on evolutionarily conserved and readily observable viral characteristics informs the relative risks posed by different zoonotic virus species, and identifies optimal targets for research and surveillance efforts.

## Methods

### Data

We compiled data on all viruses suspected to infect humans and candidate predictive features associated with each viral species. Our list was initially derived from the viral pathogens listed in GIDEON [40], ViralZone [41] and the Virus Pathogen Resource (ViPR) [42]. We further supplemented this list by searching the literature for publications describing additional viruses which have had genetic material isolated from humans, obtaining a total of 224 viral species recognized by the International Committee on Taxonomy of Viruses [43]. We also extracted information from the literature on a variety of biological features, including the genetic and structural attributes, animal hosts, arthropod vector status, and human tissue presence of each virus, and encoded this data as 19 predictor variables (S1 Table). Because their histograms showed highly skewed distributions, we log-transformed the variable for number or genome segments. To measure the influence of taxonomic grouping on transmission ability, we included each unique family and genus (using the taxonomic groupings recognized by the International Committee on Taxonomy of Viruses [43]) represented among our list of human viruses as 104 separate binary variables, for a total of 123 predictor variables. This dataset contains no missing or incomplete values (100% data coverage), and to our knowledge is more extensive in the number of viruses and viral features than past studies of the association between pathogen traits and transmission ability [18].

Based on published epidemiological information, we assigned a binary response variable to indicate whether or not there is evidence of human-to-human transmission for each virus. Our operational definition of “transmissible virus” is a pathogen which has spread via direct contact with an infectious human, as well as viruses which may be indirectly transmitted between humans through an intermediate arthropod vector or environmental source (i.e., transmission through the fecal-oral route or following contact with surface fomites). This definition does not encompass infection resulting from organ transplantation.

Cross-reactivity between antigenically related viral species can produce false positives in serological tests, so we only used the 224 virus species that have had genetic material isolated from humans to perform our analysis. All exploratory analysis and data transformations were conducted in R [44].

### Analysis

Of the 224 virus species confirmed to infect humans, we randomly selected 80% and 20% to create the training and testing sets, respectively. We repeated this process to create 40 unique data-splits, and built our primary model ensemble by fitting a single model to each unique training set using the gradient boosting machine (GBM). The GBM fits a boosted regression tree comprising a sequence of decision trees [19, 20]. Within each tree, predictors are associated with the response (here, a binary indicator of human-to-human transmission) by recursively breaking down the total pool of training observations by randomly selected splitting variables. At each step (or “split”), groups become smaller and more homogeneous. After a specified number of splits, the mean response of each group is recorded and assigned to the associated terminal node. This process is iterated to create a set of thousands of trees. Our models were built with 5500 trees and specify a Bernoulli error distribution for the binary response variable. We applied ten-fold cross validation during the fitting process to prevent overfitting. To further investigate the effect of phylogeny on transmissibility, we built and analyzed a second ensemble of 40 models that did not include any taxonomy variables. All partitioning, model building and subsequent analysis was done in R [44] using the GBM package [45].

The structure of these tree ensembles was analyzed to gain insight into the relationships between predictor and response variables. To determine the relative contribution of each predictor to classification performance, we computed relative influence scores using Friedman’s algorithm [46]. For each predictor variable in a given model, this algorithm sums the reduction in error across all nodes in the collection of trees that use the variable for splitting within a single model. These raw influence scores are then normalized as percentages and the average and standard deviation of scores across all 40 models in the ensemble were derived. We also constructed partial dependence plots, which show the marginal impact of individual predictors on the model response by integrating over the influence of all other variables [19]. The plots that we display in Fig 3 show the median effect across all 40 models in the primary ensemble, with corresponding 95% confidence intervals.

To evaluate classification performance, we computed AUC scores for each model on its associated testing and training partitions, and used these scores to derive the mean training and testing AUC of the ensemble.

A central premise of our analysis is that our dataset may contain viruses that have an unobserved ability to transmit between humans. These viruses are conservatively designated as “non-transmissible” in our analysis to denote that human-to-human transmission has not been observed, and also to minimize type II error (the error associated with classifying a human transmissible virus as unable to transmit between humans). This designation is analogous to the treatment of presence-absence data in ecology [47], and case-control data with contaminated controls in econometrics [48]. The statistical literature on this problem shows that while poorly calibrated probability estimates may be a consequence of contaminated controls, the ratio of probabilities for pairs of viruses are not affected [47, 48]. Thus, AUC scores and the rank ordering of virus species by their predicted response are not affected by the discrepancy either.

To investigate the differences in predictive performance between our tree-based machine learning approach and more rigid logistic regression models, we fit a comparable generalized linear model (GLM) to each of the 40 train-test partitions. On average, testing AUC was higher in the primary GBM models than the primary GLM models by 0.149. When evaluated by a paired Wilcoxon signed-rank test, this provides statistically significant evidence at p < 0.00001 that the median of the distribution of testing AUC scores is greater for primary GBM models than primary GLM models. When taxonomy variables were excluded from models, testing AUC was higher in GLM models by an average of 0.001 relative to GBM models, a statistically insignificant difference (p = 0.9808 under a Wilcoxon signed-rank test). These results indicate that the predictive performance of our machine learning methodology is equivalent to or greater than that of parametric logistic regression models in this situation.

## Supporting information

S1 Fig Variable partial dependence plots, supplementary models with study effort. Partial dependence plots show how the model-predicted probability that a virus is able to spread between humans is affected by individual viral traits when the effects all other predictors are controlled for. These models include the log10-transformed number of PubMed citations for each virus species as a predictor variable. The relationships between predictors and the transmission response are not meaningfully changed from those in our primary model, which does not include a study effort predictor (Fig 3). This suggests that study effort is not a confounder of variable relationships in our models.
(TIFF)

S2 Fig Variable partial dependence plots, supplementary models with a modified response definition. Partial dependence plots show how the model-predicted probability that a virus is able to spread between humans is affected by individual viral traits when the effects all other predictors are controlled for. In these models, we modified our definition of the response variable such that viruses that require an arthropod vector to spread between humans are relabeled as “non-transmissible” The relationships between predictors and the transmission response are not meaningfully changed from those in our primary model, in which the “transmissible” response group includes viruses that exclusively pass between humans indirectly through arthropod vectors (Fig 3). This indicates that our decision to not differentiate between direct transmission and indirect vector-borne transmission in the response variable did not significantly affect the trait-profile of transmissible virus we present in this study.
(TIFF)

S3 Fig Family characteristics of false-negative virus species. Each point represents a virus family that contains one or more species known to infect humans. Points represent the families of viruses included in our dataset (those known to infect humans). Red points are the 5 virus families containing the ten known-transmissible species with the lowest model-predicted transmission probability (Fig 2). These families contain relatively high numbers of individual virus species known to infect humans, few of which are known to be capable of human-to-human transmission.
(TIFF)

S1 Table Descriptions of the predictor variables included in our models. (XLSX)

S2 Table Information on the parameters, AUC scores, and variable relative influence scores of the primary and secondary models. (XLSX)

S3 Table A ranking of virus species by their mean predicted response probability in the primary GBM model ensemble. Viruses for which there is evidence of human-to-human transmission are given a value of 1 in the column *Actual*.*Response*, while viruses which are no known to be transmissible have a value of 0.
(CSV)
