# Supplementary figures and images for: Transmissibility of emerging viral zoonoses
Source: PLoS One. 2018 Nov 7;13(11):e0206926. doi: 10.1371/journal.pone.0206926 (PMC6221319; doi:10.1371/journal.pone.0206926)

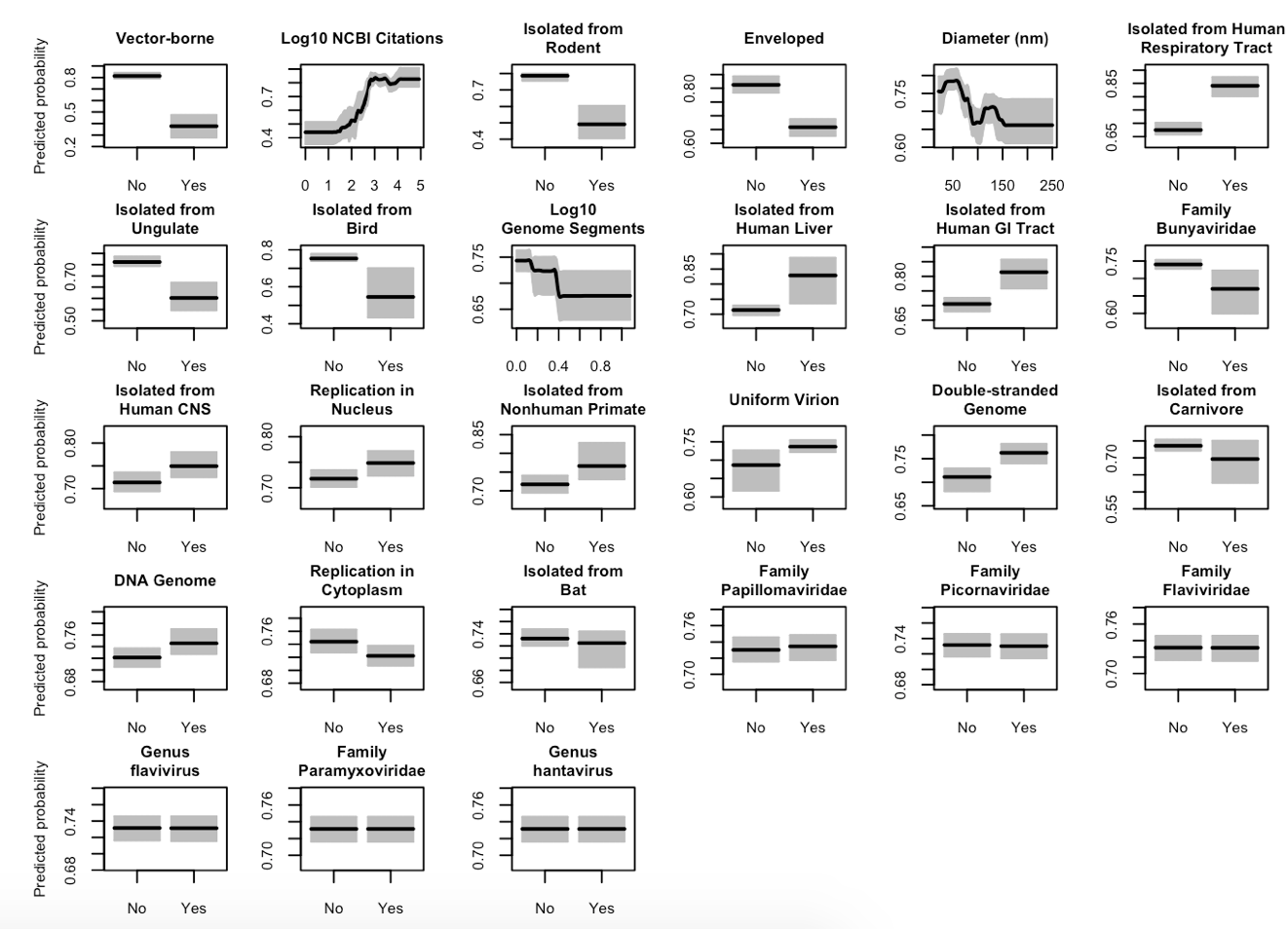

Supplement: S1 Fig — Partial dependence plots show how the model-predicted probability that a virus is able to spread between humans is affected by individual viral traits when the effects all other predictors are controlled for. These models include the log10-transformed number of PubMed citations for each virus species as a predictor variable. The relationships between predictors and the transmission response are not meaningfully changed from those in our primary model, which does not include a study effort predictor (Fig 3). This suggests that study effort is not a confounder of variable relationships in our models. (TIFF) [file pone.0206926.s001.tiff]

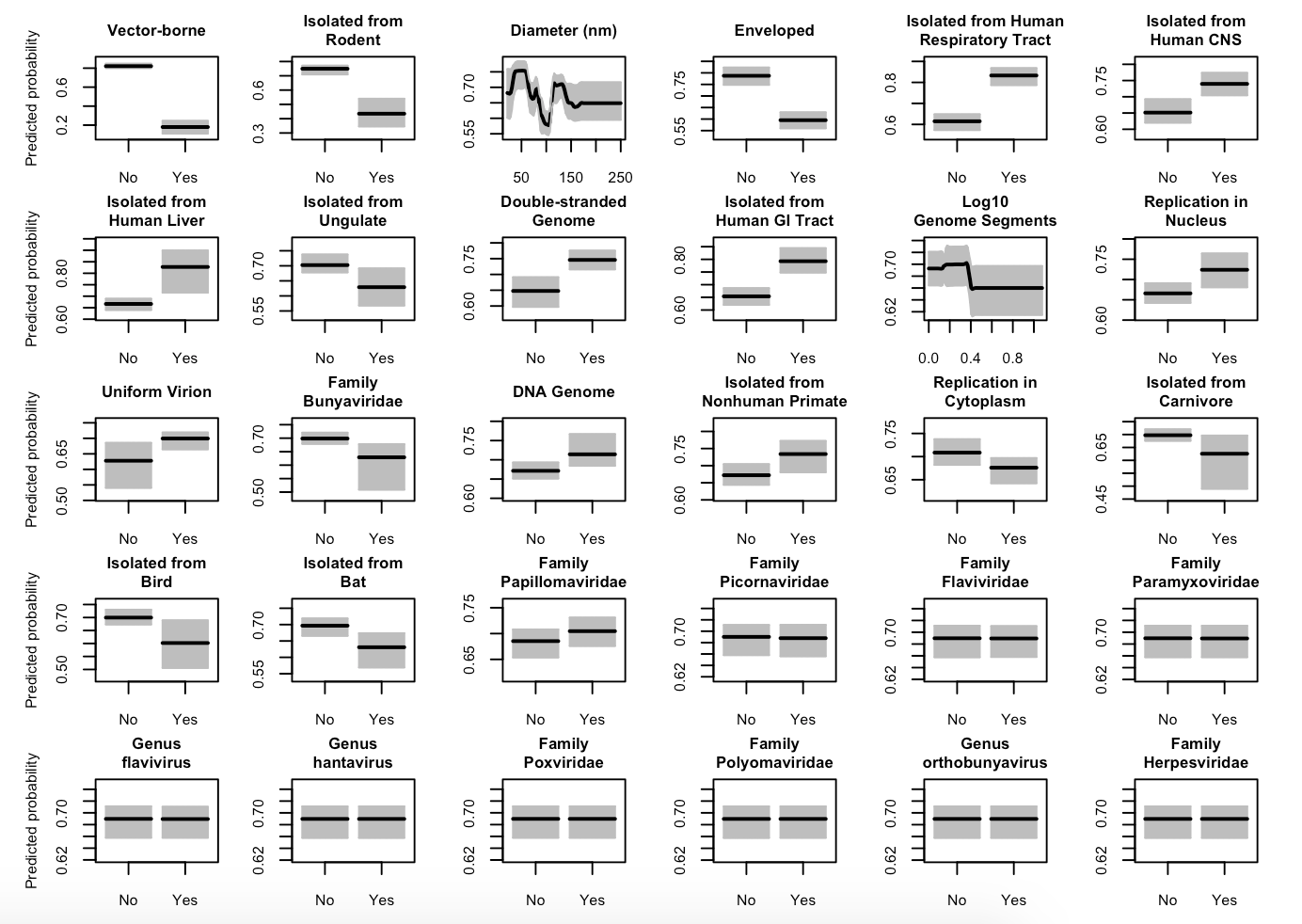

Supplement: S2 Fig — Partial dependence plots show how the model-predicted probability that a virus is able to spread between humans is affected by individual viral traits when the effects all other predictors are controlled for. In these models, we modified our definition of the response variable such that viruses that require an arthropod vector to spread between humans are relabeled as “non-transmissible” The relationships between predictors and the transmission response are not meaningfully changed from those in our primary model, in which the “transmissible” response group includes viruses that exclusively pass between humans indirectly through arthropod vectors (Fig 3). This indicates that our decision to not differentiate between direct transmission and indirect vector-borne transmission in the response variable did not significantly affect the trait-profile of transmissible virus we present in this study. (TIFF) [file pone.0206926.s002.tiff]

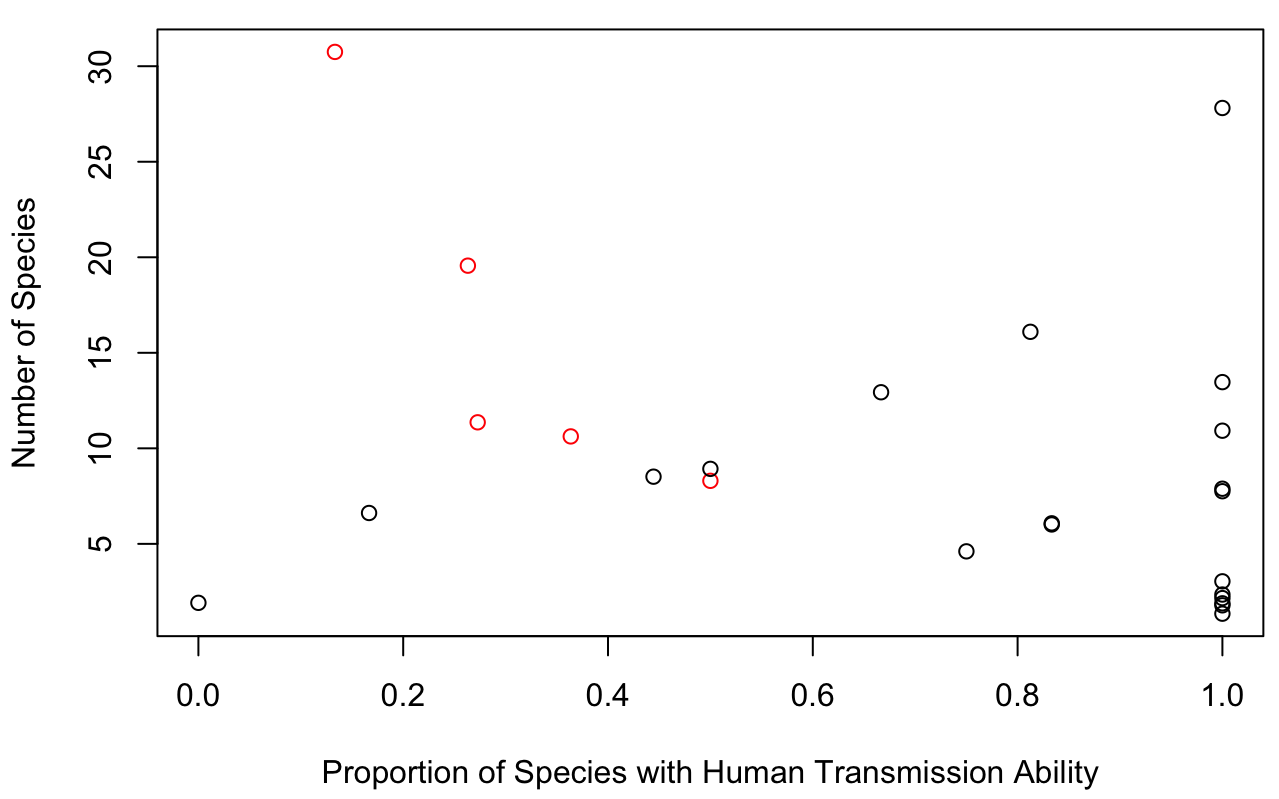

Supplement: S3 Fig — Each point represents a virus family that contains one or more species known to infect humans. Points represent the families of viruses included in our dataset (those known to infect humans). Red points are the 5 virus families containing the ten known-transmissible species with the lowest model-predicted transmission probability (Fig 2). These families contain relatively high numbers of individual virus species known to infect humans, few of which are known to be capable of human-to-human transmission. (TIFF) [file pone.0206926.s003.tiff]
